# Long-term outcome of renal cell carcinoma in patients with HIV who undergo surgery

**DOI:** 10.1186/s12879-022-07592-z

**Published:** 2022-07-09

**Authors:** Liang Chen, Menghua Wu, Xin Zheng, Yu Zhang, Jimao Zhao

**Affiliations:** 1 Department of Urology and Lithotripsy Center, Peking University People’s Hospital, Beijing, China; 2 Department of Urology, Beijing Youan Hospital, Capital Medical University, Beijing, China; 3 Department of Urology, Beijing Friendship Hospital, Capital Medical University, Beijing, China

**Keywords:** People living with HIV, Renal cell carcinoma, Prognosis, Risk factors, Overall survival, Progression-free survival, Risk factor

## Abstract

**Background:**

People living with HIV (PLWH) have a higher risk for cancer compared to the general population. The prevalence of renal cell carcinoma (RCC) in PLWH has gradually increased in recent years, but relevant data on outcomes after surgery are scarce. We thus evaluated long-term outcomes after surgery in RCC patients with and without HIV.

**Methods:**

This retrospective study included 67 patients with RCC, both HIV positive or negative, who underwent surgical treatment in our hospital between January 2012 and January 2021. Demographic details, clinical data, and cancer status were collected. We set the day of surgery as the baseline. The co-primary end points in this time-to-event analysis were overall survival and progression-free survival. We used a multivariate Cox model to compare the prognosis of PLWH and HIV-negative patients and present Kaplan–Meier curves for the co-primary end points.

**Results:**

Of 261 consecutive patients, 18 patients who forwent treatment before surgery, 133 cases with incomplete data, 16 patients classified as clinical stage IV, 11 PLWH patients did not received antiretroviral therapy and 16 patients with metastasis were excluded from the main analysis. Of the remaining 67 patients, 33 individuals had HIV and the other 34 did not. The median overall survival was 74.9 months (95% confidence interval [CI] = 64.6 to 85.2) in PLWH and 96.4 months (95% CI = 90.0 to 102.9) in the HIV-negative group. Progression-free survival was 66.4 months (95% CI = 53.5 to 79.3) and 90.6 months (95% CI = 81.1 to 100.1), respectively. RCC patients with HIV who underwent surgery had a shorter survival time (hazard ratio [HR] = 2.8, 95% CI = 1.1 to 7.0, *p* = 0.016) and an increased incidence of tumor progression (HR = 2.7, 95% CI = 1.1 to 6.8, *p* = 0.028). Univariate and multivariate Cox regression analyses showed that a lower ratio of CD4^+^ T cells to CD8^+^ T cells (adjusted odds ratio = 0.092, 95% CI = 0.01 to 0.70, *p* = 0.022) was associated with worse survival among PLWH.

**Conclusion:**

In this retrospective analysis of RCC patients who underwent surgery, PLWH had worse overall survival and shorter progression-free survival compared to HIV-negative cases.

**Supplementary Information:**

The online version contains supplementary material available at 10.1186/s12879-022-07592-z.

## Highlights

First Chinese study of HIV and RCC.

Prognostic risk factor in PLWH with RCC.

**Supplementary Information:**

The online version contains supplementary material available at 10.1186/s12879-022-07592-z.

## Introduction

At the end of 2017, there were 758,610 people living with HIV/AIDS in China, of whom 134,512 had been newly diagnosed that same year [1]. The advent of antiretroviral therapy (ART) has led to decreased mortality from opportunistic infectious diseases. This has improved survival and reduced the frequency of HIV-associated illnesses and complications of AIDS in people living with HIV (PLWH) [2, 3], who are at elevated risk for developing several types of cancer. Since the 1990s, the U.S. Centers for Disease Control and Prevention has defined Kaposi sarcoma, non-Hodgkin’s lymphoma, and cervical cancer as AIDS-defining cancers [4]. In an age without widespread use of ART, active management of other non-AIDS-defining cancers (NADCs) was less common, for the life expectancy of PLWH was relatively short [5]. As survival has improved [6–8], NADCs have consistently been reported, as reflected in the higher risk for cancer in PLWH compared to the general population [9].

In a large meta-analysis of seven population-based HIV cancer studies that involved more than 400,000 HIV-positive patients, the standardized incidence ratio for renal cell carcinoma (RCC) was 1.50 (95% confidence interval [CI] = 1.23 to 1.83) in PLWH [10]. However, most data on risk for NADCs derive from Western countries; relatively few studies have addressed this risk in Asian people. Surgery is the preferred option for treating RCC, and the prevalence of laparoscopic surgery and nephron-sparing surgery is gradually increasing [11]. Yet it remains unknown whether PLWH who undergo surgery for RCC exhibit a different clinical outcome compared to HIV-negative individuals with RCC. Clinical features such as level of serum albumin and tumor size have been reported as risk factors affect renal cell carcinoma outcomes in general population[12–16]. Moreover, the ratio of CD4^+^ T cells to CD8^+^ T cells (CD4^+^/CD8^+^ ratio) was also recently reported to be a prognostic marker for other NADCs [17]. We wondered whether PLWH with NADCs have worse outcomes than HIV-negative population and whether the factors above influence their prognosis. Thus, we studied RCC patients referred to our hospital to compare long-term overall survival and progression-free survival between PLWH and patients without HIV and explore potential factors influencing outcomes.

## Methods

### Patients and characteristics

This study included PLWH and HIV-negative patients with RCC who presented at the Department of Urology in Beijing Youan Hospital from January 2012 to December 2014 and who were followed by telephone until January 2021. Our study was approved by the Youan institutional ethics committee, and patients’ clinical data came from electronic health records in the hospital information system. All patients remained anonymous, and informed consent was waived.

The inclusion criteria were as follows: (1) Patients were 18 years of age or older, had localized RCC, and could adopt surgery; (2) the PLWH group met the diagnostic criteria for adult HIV/AIDS developed by the U.S. Centers for Disease Control and Prevention; (3) procedures complied with the 2019 revised RCC tumor-node metastasis staging published by the American Joint Committee on Cancer [18]; (4) patients had a postoperative pathological diagnosis and clinical stage I–III renal cancer; and (5) PLWH had received ART for at least 4 months prior to surgery.

The exclusion criteria were as follows: Patients (1) did not have ART or did not keep scheduled visits, (2) had a first-time diagnosis of RCC and an existing metastatic tumor, (3) did not undergo surgery, or (4) had incomplete data.

Data on demographic features and clinical characteristics were retrospectively collected, including age, gender, CD4^+^ T cell count and CD4^+^/CD8^+^ ratio, laboratory serum tests, pathologic features, Glasgow Prognostic Score (GPS), Charlson Comorbidity Index (CCI), Karnofsky performance status (KPS), and Leibovich score. Overall survival and progression-free survival (according to Response Evaluation Criteria in Solid Tumors, version 1.1, by CT from our hospital or from local hospitals) were calculated from the date of surgery to the time of death or last follow-up.

The GPS was calculated from the level of albumin and C-reactive protein as previously reported [19]. The CCI was scored according to 19 preoperative comorbidities [20]. The KPS was used to quickly quantify the general health of the patient and his or her ability to perform daily activities [15, 21]. The Leibovich score 2003 is a scoring algorithm based on these features that can be used to predict disease progression after surgery for clinically localized RCC [22].

### End point

The primary end point was calculated from the day of surgery to death or tumor progression. If patients died after tumor progression, the timing of the primary end point was defined as the length of time between the date of surgery and the progression of the tumor. Disease progression was based on investigator review and Response Evaluation Criteria in Solid Tumors, version 1.1. We censored patients who were lost to follow-up or who were last known to be alive at data cutoff.

### Data and analysis

We estimated that in a test of significance, a sample size of 70 patients would provide 75% power to detect a hazard ratio (HR) for overall survival of 0.5 with a favorable outcome in HIV-negative patients over PLWH at a two-sided alpha level of 0.05 (Additional file 1: Fig. S1).

Statistical analyses were performed in SPSS 22.0 (IBM, Armonk, NY, USA). Significant differences among groups were determined by analysis of variance, and differences between two independent groups were evaluated with Student’s *t* test. We also estimated HRs and 95% CIs between groups using Cox regression models. Dynamic change in overall and progression-free survival is presented via Kaplan–Meier curves. Cox regression was also used to analyze correlations between patient characteristics and survival. Variables that were significant at *p* < 0.05 in the univariate analysis and those previously considered critical were considered meaningful and were included in the Cox regression analyses.

## Results

**Fig. 1:**
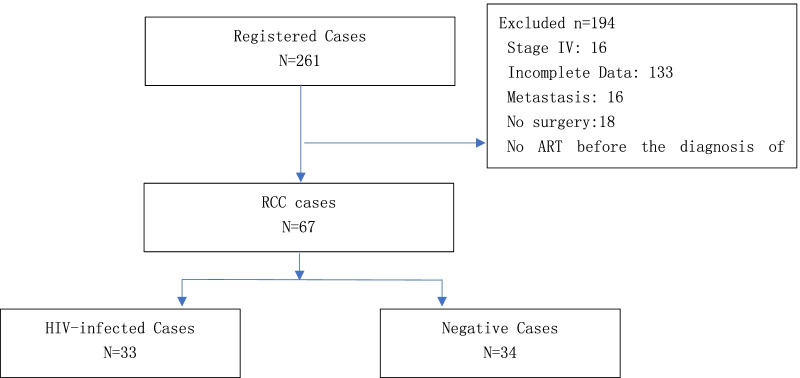
Flow chart of data collection and analysis

Of the 3259 patients who were managed by the Department of Urology in Beijing Youan Hospital between January 2012 and December 2014, 261 received a diagnosis of RCC. As expected, RCC patients with metastasis had a worse survival time compared to patients without metastasis (Additional file 2: Fig. S2). Patients with early-stage cancer (I–III) underwent surgery. Ultimately, 67 patients met the study criteria: 33 PLWH and 34 HIV-negative patients (Fig. 1). Demographic data and clinical characteristics are presented in Table 1. Most RCC was clear cell carcinoma. Creatinine (77.1 ± 18.8 vs. 85.7 ± 12.9, *p* = 0.032), hemoglobin (113.6 ± 12.6 vs. 139.3 ± 20.8, *p* = 0.001), and the CD4^+^/CD8^+^ ratio (0.68 ± 0.40 vs. 0.86 ± 0.33 *p* = 0.041) were lower among PLWH. No other significant differences in clinical features were found between the groups at baseline.

**Table 1:** General clinical characteristics and tumor status

Feature	Total	HIV group	Control
N	67	33	34
Age at surgery	51 ± 11	51 ± 12	50 ± 11
BMI	24.8 ± 2.6	24.4 ± 2.2	25.1 ± 2.9
Gender			
Female/Male	12/55	5/28	7/27
Creatinine	81.4 ± 16.5	77.1 ± 18.8	85.7 ± 12.9
eGFR	91.33 ± 17.29	94.69 ± 19.69	88.06 ± 14.15
CRP	10.92 ± 2.67	7.45 ± 3.78	14.94 ± 30.05
Albumin	42.5 ± 4.7	41.6 ± 4.8	43.4 ± 4.4
HGB (g/L)	126.6 ± 21.6	113.6 ± 12.6	139.3 ± 20.8
Tumor Size	4.42 ± 2.51	4.52 ± 2.95	4.33 ± 1.95
CD4 cell count(cell/)	523.3 ± 223.1	401.7 ± 210.1	641.3 ± 166.2
CD4/CD8 ratio	0.77 ± 0.37	0.68 ± 0.40	0.86 ± 0.33
Clinical Stage			
cT1	34	16	18
cT2	25	13	12
cT3	8	4	4
Pathological Stage			
pT1	46	24	21
pT2	12	5	7
pT3	10	4	6
Side			
Left/Right	43/24	19/14	24/10
GPS			
0	40	20	20
1	24	11	13
2	3	2	1
Histology, n (%)			
Clear cell tumor	58	27	31
Chromophobe cell tumor	6	5	1
Other	3	1	2
Positive HBsAg, n (%)	4	3	1
Positive anti-HCV Ab, n (%)	3	1	2
CCI	7.58 ± 1.75	6.85 ± 1.18	8.69 ± 1.93
Baseline ART regimen			
PI-based regimen	14	14	-
NNRTI-based regimen	18	18	-
INSTI-based regimen	1	1	-
Grade			
1	33	21	16
2	24	8	13
3	9	4	6
Surgical approach			
Partial resection	20	7	13
Radical nephrectomy	47	26	21
Bleeding(mL)	135.7 ± 77.4	114.0 ± 51.8	169.2 ± 98.5
Operating Time(h)	1.3 ± 0.4	1.2 ± 0.4	1.4 ± 0.5
Length of stays	11.7 ± 3.8	11.8 ± 3.8	11.6 ± 3.9
KPS	90.3 ± 12.8	91.8 ± 13.1	88.8 ± 12.5
Leibovich Score 2003	1.22 ± 1.62(0–6)	1.27 ± 1.62	1.18 ± 1.66

*BMI* Body mass index, *eGFR* estimated glomerular filtration rate, *CRP* C-reactive protein, *HGB* hemoglobin, *CCI* Charlson comorbidity index, *GPS* Glasgow Prognostic Score, *ART* active antiretroviral therapy, *PI* Protease inhibitor; *NNRTI* non-nucleoside reverse transcriptase inhibitors, *INSTI* integrase strand transfer inhibitor, *KPS* Karnofsky performance status

**Fig. 2:**
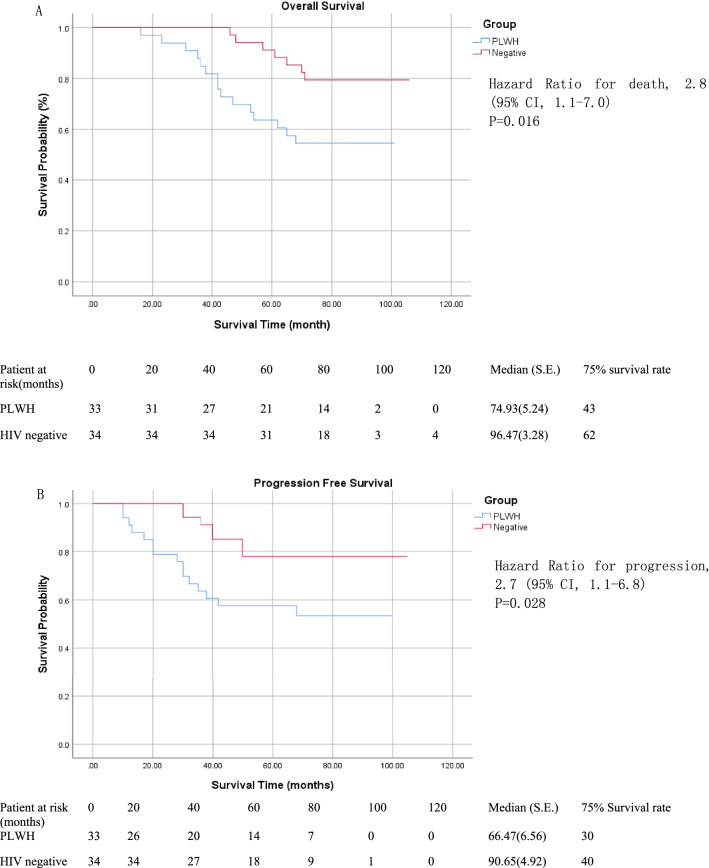
Kaplan–Meier analysis of overall and progression-free survival for the PLWH group (blue line) and HIV-negative group (red line). Shown are Kaplan–Meier estimates of overall survival **A** and progression-free survival **B** according to Response Evaluation Criteria in Solid Tumors, version 1.1. *CI* confidence interval

Kaplan–Meier curves of overall survival and progression-free survival are shown in Fig. 2. During a median follow-up of 73.5 months, the median overall survival time was 74.9 months (95% CI = 64.6 to 85.2) in the PLWH group and 96.4 months (95% CI = 90.0 to 102.9) in the HIV-negative group. Progression-free survival was 66.4 months (95% CI = 53.5 to 79.3) and 90.6 months (95% CI = 81.1 to 100.1), respectively. RCC patients with HIV who underwent surgery had a shorter survival time (HR = 2.8, 95% CI = 1.1 to 7.0, *p* = 0.016) and a higher incidence of tumor progression (HR = 2.7, 95% CI = 1.1 to 6.8, *p* = 0.028).

Hemoglobin, serum albumin and creatinine, the CD4^+^/CD8^+^ ratio, and tumor size were entered into Cox regression analyses (Table 2). Univariate and multivariate Cox logistic regression analyses showed that the lower CD4^+^/CD8^+^ ratio (adjusted odds ratio = 0.092, 95% CI = 0.01 to 0.70, *p* = 0.022) was associated with worse survival among PLWH. The results of a bivariate analysis with scatter plot between the CD4^+^/CD8^+^ ratio and overall survival in PLWH are shown in Additional file 3: Fig. S3.

**Table 2:** Univariate and multivariate Cox regression analyses for overall survival

	Unadjusted analysis		Adjusted analysis	
Variables	Odds ratio(95% CI)	*P*-value	Odds ratio(95% CI)	*P*-value
CD4^+^/CD8^+^ ratio	0.086 (0.010–0.683)	0.029	0.092 (0.011–0.706)	0.022
Albumin	0.970 (0.907–1.038)	0.381	0.931 (0.837–1.036)	0.190
Hemoglobin	0.112 (0.976–1.156)	0.301	1.009 (0.981–1.043)	0.541
Creatinine	1.008 (0.988–1.029)	0.427	1.013 (0.988–1.038)	0.322
Tumor size	1.162 (1.014–1.423)	0.036	1.177 (1.024–1.352)	0.021

Results of the univariate and multivariate Cox regression analyses for all patients

## Discussion

In this retrospective study, we enrolled a cohort of PLWH diagnosed with RCC who were treated in our hospital, a specialized hospital for infectious diseases. After patients underwent surgery, we performed long-term follow-up by telephone until 2021. We investigated numerous clinical features, including tumor status, demographic information, and laboratory tests (e.g., creatinine, estimated glomerular filtration rate, albumin, hemoglobin, and C-reactive protein). PLWH with NADCs may be at greater risk for postoperative complications and infection than the general population and may have tumors that are larger or more advanced at diagnosis [4, 23]. Tumor size was previously shown to be closely related to outcomes in patients with RCC in the general population. Serum albumin has been used in a number of RCC studies in the general population to predict prognosis [15, 22]. However, there was no difference in tumor size or albumin at baseline or surgical complications between the two groups in this study, although there was slightly lower serum creatinine and relatively lower hemoglobin in the PLWH group. Neither creatinine nor hemoglobin has been shown to be clearly associated with RCC prognosis in studies of the general (HIV-negative) population [14, 24, 25], and are probably coincidental factors here. Compared to HIV-negative patients, PLWH had a significantly worse prognosis in terms of both overall survival and progression-free survival.

Previous studies of the relationship between HIV and tumors have mainly focused on the disparity in the incidence of cancer among people living with and without HIV. Data on the prognosis following surgery in PLWH with malignancy are limited. In a national cohort, short-term outcomes after surgical lung resection did not differ significantly between lung cancer patients with and without HIV well managed with ART medication [26]. Another study reported that HIV status did not adversely affect outcomes in African patients with head and neck cancer [27], but this conclusion was limited by the suboptimal medical treatment received by all participants in local areas. Wang et al. [23] suggested that HIV viral load and CD4^+^ T cell counts may affect postoperative outcomes or complications. In our study, even with ART treatment, the CD4^+^/CD8^+^ ratio varied in the PLWH group. Moreover, our study demonstrates an association between a lower CD4^+^/CD8^+^ ratio and worse overall survival.

The main factor affecting the prognosis of PLWH may be the onset of HIV. PLWH who receive ART have a similar, but not equal, prognosis to that of uninfected individuals. In our study, the CD4^+^ T cell count in PLWH was similar to that in the HIV-negative group. Previous studies using the CD4^+^/CD8^+^ ratio as a surrogate marker of immune status have shown that it is independently associated with NADCs and mortality in PLWH, whereas CD4^+^ T cell counts alone do not predict the risk of survival in PLWH with NADCs [17, 28, 29]. Mussini et al. conducted a cohort study to investigate the association between the CD4^+^/CD8^+^ ratio and morbidity in PLWH on ART and found that a CD4^+^/CD8^+^ ratio < 0.5 can identify patients who require more intensive cancer prevention or screening [28]. These findings further validate prior recommendations advocating surgery for PLWH regardless of the CD4^+^ T cell count. The CD4^+^/CD8^+^ ratio seems to be an important prognostic marker in PLWH, and in our study a lower CD4^+^/CD8^+^ ratio in PLWH may have predicted worse survival.

We have not found any study that has directly compared the prognosis of PLWH and HIV-negative patients with RCC. Whether the HIV itself or the tumor itself affects the prognosis is unclear. Although in our study the two groups have a similar pathological stage, general status, KPS, GPS, and CCI, PLWH were still prone to worse overall survival and progression-free survival. These findings further validate prior recommendations advocating surgery for PLWH upon an earlier pathological stage [4, 11].

In the past decade, understanding of the determinants of the ontology of RCC has come a long way. The loss of chromosome 3p is a common genetic abnormality confirmed in both hereditary and sporadic RCC. Tumor genomic features can affect distinct immune phenotypes. For example, PBRM1 mutations are associated with reduced infiltration of T cells; however, whether they are associated with RCC in PLWH remains unclear [30]. The mechanisms behind HIV with RCC may be more complex, and future research is needed on their interactive effects.

Several limitations of our study should be acknowledged. First, it was a single-center retrospective research study with a limited sample size; therefore, the generalizability of the findings to patients in other places may be limited. Second, insufficient information was available in the telephone follow-up. For example, whether socioeconomic status or HIV-related health problems was mostly responsible for the poor prognosis of PLWH is not known.

## Conclusion

In our retrospective analysis, RCC patients with HIV had both shorter overall survival and worse progression-free survival than patients without HIV. The lower CD4^+^/CD8^+^ ratio indicated a worse outcome.

## Supplementary Information

**Additional file 1: Fig S1. **The sample size calculation through PASS 21.

**Additional file 2: Fig S2.** Kaplan-Meier Analysis of Overall survival with Localized and metastasis RCC.

**Additional file 3: Fig S3. **Bivariate analysis to present relationship between CD4+/CD8+ ratio and overall survival for PLWH.

## Data Availability

The data sets generated and/or analyzed in the current study are not publicly available, as they contain protected health information, but are available from the corresponding author on reasonable request.
