# Supplementary material for: Long-term outcome of renal cell carcinoma in patients with HIV who undergo surgery
Source: BMC Infect Dis. 2022 Jul 9;22:605. doi: 10.1186/s12879-022-07592-z (PMC9270790; doi:10.1186/s12879-022-07592-z)
Supplement: Supplementary file 1 — Additional file 1: Fig S1. The sample size calculation through PASS 21. [file 12879_2022_7592_MOESM1_ESM.docx]

**
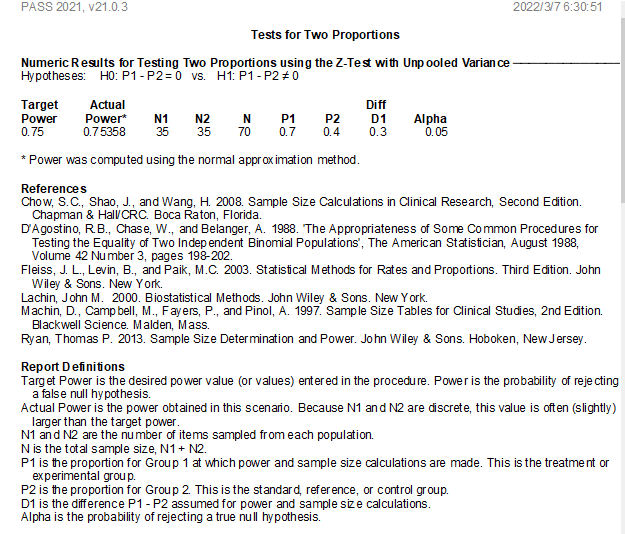
Figure S1. The sample size calculation through PASS 21**

We estimated the sample size of 70 patients in a significance of test of difference
