# Supplementary material for: Long-term outcome of renal cell carcinoma in patients with HIV who undergo surgery
Source: BMC Infect Dis. 2022 Jul 9;22:605. doi: 10.1186/s12879-022-07592-z (PMC9270790; doi:10.1186/s12879-022-07592-z)
Supplement: Supplementary file 2 — Additional file 2: Fig S2. Kaplan-Meier Analysis of Overall survival with Localized and metastasis RCC. [file 12879_2022_7592_MOESM2_ESM.docx]

**
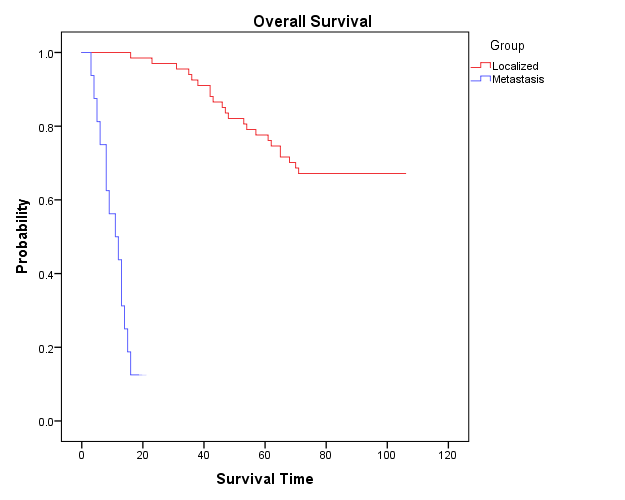
Figure S2.** **Kaplan-Meier Analysis of Overall survival with Localized and metastasis RCC.**

Shown are Kaplan–Meier estimates of overall survival in patients with localized RCC (red line, n=67) or patients with metastasis RCC (blue line, n=16).
