# Supplementary material for: Long-term outcome of renal cell carcinoma in patients with HIV who undergo surgery
Source: BMC Infect Dis. 2022 Jul 9;22:605. doi: 10.1186/s12879-022-07592-z (PMC9270790; doi:10.1186/s12879-022-07592-z)
Supplement: Supplementary file 3 — Additional file 3: Fig S3. Bivariate analysis to present relationship between CD4+/CD8+ ratio and overall survival for PLWH. [file 12879_2022_7592_MOESM3_ESM.docx]

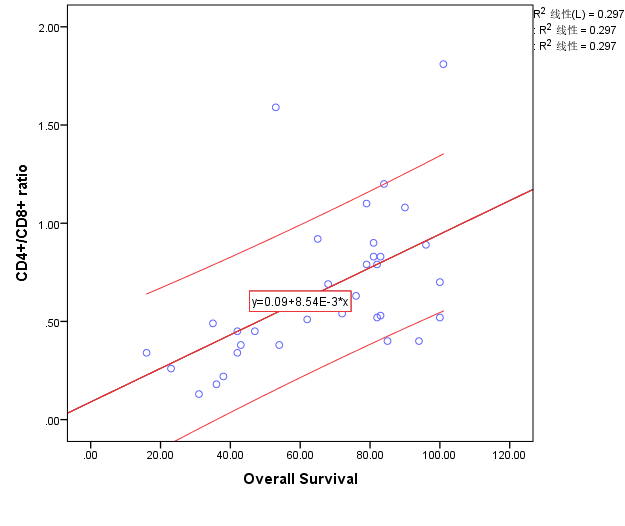
F

**Figure S3. Bivariate analysis to present relationship between CD4+/CD8+ ratio and overall survival for PLWH.**

The Person’s correlation coefficient is 0.545, the linear formula is y=0.05*8.54-3*x, P value = 0.001, the upper line and the lower line denotes quartile interval.
